# Tension Viscerothorax with Concomitant Pneumothorax and Associated Intrathoracic Gastric Perforation after Thoracoabdominal Stab Wound in a Resource-Limited Setting: A Case Report

**DOI:** 10.70352/scrj.cr.26-0110

**Published:** 2026-05-12

**Authors:** Amanuel Mesfin Oljira, Sinbona Ararsa Keneni, Solomon Guteta Beyene, Diriba Gebeyehu Wakesa, Rabirra Waktola Gonfa, Eliab Negusse Reda

**Affiliations:** 1 Department of Surgery, College of Medicine and Health Sciences, Ambo University, Ambo, Oromia, Ethiopia; 2 Department of Radiology, College of Medicine and Health Sciences, Ambo University, Ambo, Oromia, Ethiopia; 3 Department of Public Health, School of Medicine, College of Health Sciences, Addis Ababa University, Addis Ababa, Ethiopia

**Keywords:** diaphragmatic injury, tension viscerothorax, stab wound, pneumothorax, gastric perforation, gastrothorax, resource-limited setting, case report

## Abstract

**INTRODUCTION:**

Traumatic diaphragmatic injury after penetrating thoracoabdominal trauma is rare and can be missed when CT is not available. Tension viscerothorax can mimic tension pneumothorax and may persist despite pleural decompression.

**CASE PRESENTATION:**

A 28-year-old man suffered a knife stab to his left posterior thoracoabdomen. A 28-French left chest tube inserted at a local health center for a suspected tension pneumothorax (without imaging) did not improve the patient's condition, and later drained the gastrointestinal (GI) content. Upon arrival, chest radiography revealed a left pneumothorax with a severe mediastinal shift to the right, as well as a gastric air bubble and bowel loops in the left hemithorax. Focused assessment with sonography for trauma (FAST) showed a large left pleural fluid collection and splenorenal fluid accumulation. Nasogastric decompression aspirated air and 300 mL of GI fluid with partial improvement. Emergency laparotomy revealed a 10-cm left diaphragmatic laceration (American Association for the Surgery of Trauma grade III) with immediate multivisceral herniation, a jejunal serosal tear, and an associated through-and-through fundal gastric perforation, contaminating the pleural and peritoneal cavities. Primary repair of the gastric, jejunal, and diaphragmatic injuries, combined with lavage and revision of the chest tube, achieved source control. He was discharged on POD 8 and remained symptom-free without recurrence at 12-month follow-up.

**CONCLUSIONS:**

In resource-limited settings, persistent obstructive physiology despite tube thoracostomy, especially when accompanied by bowel sounds in the hemithorax or enteric drainage through the chest tube, should prompt suspicion for tension viscerothorax. Classical clinical findings, together with chest radiography, FAST, and early nasogastric decompression, can justify timely definitive surgery even when CT is unavailable.

## Abbreviations

AAST
: American Association for the Surgery of Trauma
BP
: blood pressure
CXR
: chest radiograph
FAST
: focused assessment with sonography for trauma
Fr
: French size
GCS
: Glasgow Coma Scale
GI
: gastrointestinal
IV
: intravenous
NGT
: nasogastric tube
TDI
: traumatic diaphragmatic injury

## INTRODUCTION

Penetrating injuries in the thoracoabdominal junction can lacerate the diaphragm and permit abdominal viscera to migrate into the negative-pressure thorax, yet the diagnosis is often delayed or missed even in well-resourced systems.^1–4)^ Acute massive herniation may cause obstructive shock (tension viscerothorax, or tension gastrothorax when gastric herniation predominates).^5)^ This overlaps clinically with tension pneumothorax; pleural decompression alone may not relieve mediastinal compression when herniated viscera are the primary space-occupying lesion, and gastrothorax may coexist with or precipitate tension pneumothorax.^6)^ In settings where a CT scan is unavailable, clinical findings and basic imaging (CXR and FAST) can guide timely definitive management.^7–9)^

## CASE PRESENTATION

A previously healthy 28-year-old man suffered a single knife stab to the left posterior thoracoabdomen at 21:00. He had no known diseases, did not take regular medications, and had no smoking or alcohol use; his family history was non-contributory. At 00:30, he reached a local health center, where the wound was dressed, and a left 28-Fr tube thoracostomy (5th intercostal space, mid-axillary line) was inserted for suspected tension pneumothorax (no imaging was available). The chest tube was connected to an underwater seal, but there was no clinical improvement, prompting referral. He arrived at Ambo University Referral Hospital at 09:00 (12h after injury) in severe respiratory distress, but he was alert (GCS 15). His BP was 87/53 mmHg, pulse rate was 120 beats/min, respiratory rate was 32 breaths/min, temperature was 38.9°C, and oxygen saturation was 91% on nasal cannula at 5 L/min. Physical examination showed a 3 × 2-cm stab wound in the 7th intercostal space at the posterior axillary line on the left posterior chest. The chest tube was functional and had drained 750 mL upon arrival; enteric fluid mixed with blood was present in the output. The trachea was deviated to the right; breath sounds on the left were markedly decreased with hyperresonance, and bowel sounds were heard in the left hemithorax. Neck veins were distended. The abdomen was flat and diffusely tender with guarding and rebound tenderness. Other system examinations were within normal limits. A timeline of events is provided separately (**Table 1**).

**Table 1:** Timeline of events

Phase	Relative day	Approximate time	Event	Location	Key details
Preoperative	Injury day	~21:00	Stab injury to left posterior thoracoabdomen	Community	Start of clinical course
Preoperative	Preop day 0 (≈3.5 h post-injury)	~00:30	Arrival at local health center; wound dressed	Local health center	No imaging performed/available
Preoperative	Preop day 0	~00:45	Left tube thoracostomy inserted (28 Fr) for presumed tension pneumothorax	Local health center	5th intercostal space, mid-axillary line; underwater seal; no documented improvement
Preoperative	Preop day 0	~03:30	Departure/referral	En route	Transfer time ~6 h; received 2 bags LR; nasal O_2_ 5 L/min; no deterioration reported
Preoperative	Preop day 0 (≈12h post-injury)	~09:00	Arrival to referral hospital and ED assessment	Referral hospital (ED)	Severe respiratory distress/obstructive physiology; chest tube swinging; enteric drainage noted; 750 mL drained by arrival
Preoperative	Preop day 0	Shortly after arrival	CXR + FAST performed	ED	CXR: left pneumothorax, severe mediastinal shift, intrathoracic gastric bubble/bowel loops; FAST: large left pleural and splenorenal fluid; no pericardial fluid
Preoperative	Preop day 0	~09:20	NGT inserted (difficult) after CXR	ED	Aspirated air + ~300 mL enteric content; partial physiologic improvement
Operative	POD 0	10:00	Emergency midline laparotomy	Operating room	10-cm left diaphragmatic laceration with multivisceral herniation; through-and-through fundal gastric perforation; lavage; primary repairs; diaphragm closed with 0 Prolene; chest tube revised after closure (28 Fr)
Postoperative	POD 0–POD 1	—	ICU ventilation and stabilization	ICU	Intubated intraop; ventilated for ~1 day postop
Postoperative	POD 2	—	Extubation and step-down	ICU → ward	Extubated on ICU day 2; briefly on nasal O_2_ 5 L/min then off O_2_; transferred to surgical ward same day; chest physiotherapy started POD 2
Postoperative	POD 0–POD 2	—	NGT continued postop	ICU/ward	NGT kept for 2 days; removed after flatus, reduced distension, and output <200 mL/24h for 2 consecutive days
Postoperative	POD 4 (approx.)	—	Oral feeding initiated	Ward	Oral feeding started 2 days after NGT removal
Postoperative	POD 6	—	Chest tube removed	Ward	Removed after <200 mL/24h output for 2 consecutive days + CXR re-expansion + good air entry
Postoperative	POD 8	—	Discharged home	Ward/discharge	Discharged without complications
Follow-up	1, 3, 6, 12 months	—	Outpatient follow-up	Clinic	Symptoms resolved; no recurrence at 12 months; returned to work at ~2 months

CXR, chest radiograph; ED, emergency department; FAST, focused assessment with sonography for trauma; Fr, French size; h, hours; intraop, intraoperative; LR, lactated Ringer’s; NGT, nasogastric tube; preop, preoperative

### Differential diagnosis, investigations, and treatment

#### Differential diagnosis

The main differential diagnoses were tension pneumothorax (given severe respiratory distress, tracheal deviation, and hypotension); massive hemothorax or tension hemopneumothorax; TDI with visceral herniation causing tension viscerothorax with or without associated pneumothorax; and cardiac tamponade, which was considered in the setting of obstructive shock, was excluded by FAST demonstrating no pericardial fluid.

**Fig. 1:**
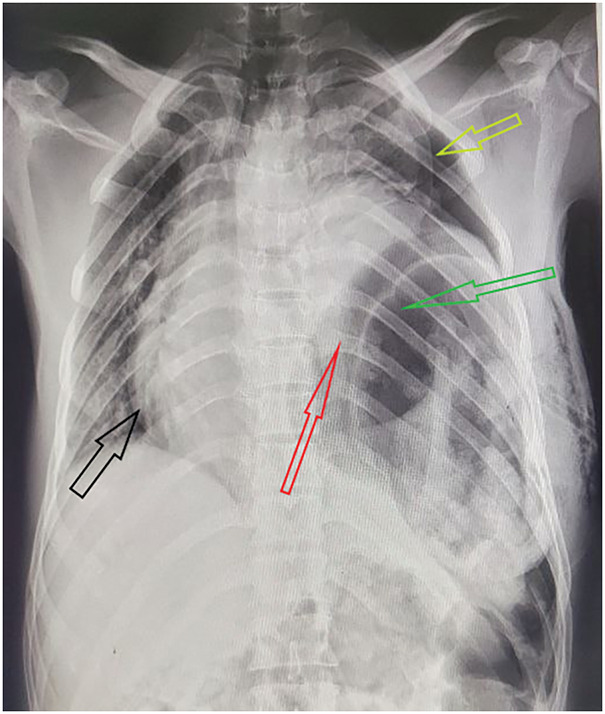
Anteroposterior CXR demonstrating left pneumothorax with collapse of the left lung (yellow arrow), severe rightward mediastinal shift (black arrow), loss of the left diaphragmatic contour, intrathoracic gastric bubble and bowel loops in the left hemithorax (red and green arrows), and the pre-existing left chest tube *in situ*. No rib fracture is visible. CXR, chest radiograph

Laboratory tests revealed leukocytosis (white blood cell count 21.3 × 10^3^/L, neutrophils 94%), hemoglobin of 11.2 g/dL, serum creatinine of 0.9 mg/dL, blood urea nitrogen 18 mg/dL, alanine transaminase 24 U/L, aspartate transaminase 30 U/L, and alkaline phosphatase 114 U/L. Chest radiography showed left pneumothorax with left lung collapse, severe mediastinal shift to the right, loss of the left diaphragmatic outline, and left intrathoracic gastric air bubbles and intestinal loops, but no rib fractures (**Fig. 1**). FAST performed by the surgical team showed a large amount of fluid in the left pleural space and splenorenal recess, but no pericardial fluid accumulation. The patient received 2 L of IV Ringer’s lactate as a bolus. An NGT was inserted with difficulty, which immediately aspirated air and 300 mL of GI content, resulting in partial improvement in the patient's subjective respiratory distress and vital signs. IV antibiotics (ceftriaxone 1g IV and metronidazole 500 mg IV) were started in the emergency department 45 min before skin incision. Urine output was 1.5 mL/kg/h before emergency laparotomy. CT was not available at Ambo University Referral Hospital.

**Fig. 2:**
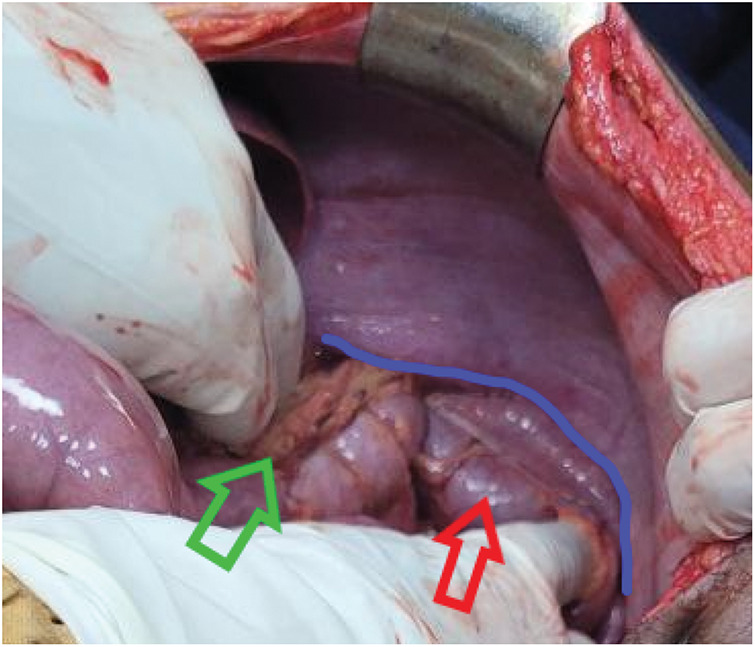
Intraoperative view showing a large left diaphragmatic laceration (blue line) with herniation of the stomach, transverse colon (red arrow), spleen, jejunum, and omentum (green arrow) into the thoracic cavity.

**Fig. 3:**
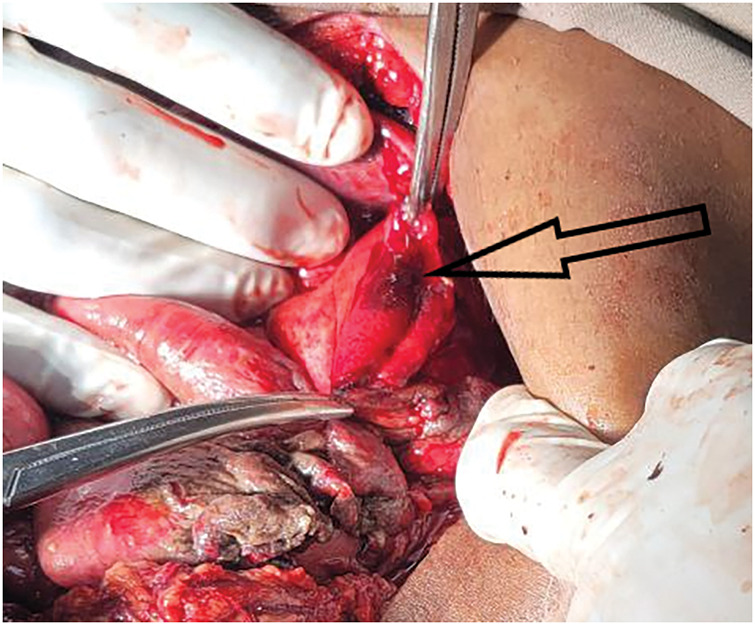
Intraoperative view showing the through-and-through perforation of the gastric fundus (black arrow) identified after reduction of the herniated viscera.

Emergency midline laparotomy, performed 1h after arrival, showed a 10-cm anterolateral defect in the left hemidiaphragm, with stomach fundus, greater omentum, superior splenic pole, left third of the transverse colon, and approximately 20 cm of mid-jejunum herniating into the thorax (**Fig. 2**). About 2.5L of enteric fluid were found in the pleural cavity and 300 mL in the peritoneum, with no foul smell present. A 5 × 1-cm through-and-through gastric fundal perforation, situated on the greater curvature 7 cm from the gastroesophageal junction (**Fig. 3**), a 2-cm serosal tear on the antimesenteric side of the mid-jejunum, and a 1-cm capsular laceration in the upper spleen were discovered. There was no active bleeding from the injured organs. Herniated viscera were reduced; pleural and peritoneal cavities were irrigated through the diaphragmatic defect with normal saline. Freshening of the edges of the gastric perforation and closure were performed in 2 layers with Vicryl 3-0 (Ethicon, Raritan, NJ, USA) and reinforced with a pedicled omental patch. The jejunal serosal tear was repaired with interrupted Lembert sutures using Vicryl 3-0. The diaphragm was closed in an interrupted fashion using Prolene 0 (Ethicon) without mesh. The chest tube was revised in the operating room following closure of the diaphragm, and the abdomen was then closed in layers. The hemodynamic status of the patient remained within normal limits during the operation (BP range 125/73–110/65 mmHg; pulse rate 108–112 beats/min).

He was given ceftriaxone 1g IV twice daily, along with metronidazole 500 mg IV 3 times daily for 7 days postoperatively. For pain relief, he received tramadol 50 mg IV 3 times daily and diclofenac 75 mg intramuscularly once daily for 7 days; there were no reported adverse effects, and the treatment was well tolerated. He was placed on a ventilator in the ICU for 1 day, was extubated on the second day of his ICU stay, and was moved to the general ward on the same day. The NGT was kept in place for 2 days following surgery, and oral feeding was started 2 days after the NGT was removed. Chest physiotherapy began on POD 2, and the chest tube was removed on POD 6 following low drainage and radiographic evidence of lung re-expansion. He was discharged from the hospital on POD 8 with counseling on wound care, feeding, ambulation, and return precautions. Follow-up at 1 month, 3 months, 6 months, and 1 year (with history, physical examination, laboratory tests, and CXR) showed no complications, and he returned to work 2 months after discharge.

## DISCUSSION

Tension viscerothorax is a high-risk mimic of tension pneumothorax. The key educational value of this case lies not simply in the radiographic diagnosis but in the bedside recognition of persistent obstructive physiology despite chest tube decompression, bowel sounds in the hemithorax, enteric drainage through the chest tube, and partial physiologic improvement after nasogastric decompression. Together, these findings redirected management toward urgent laparotomy in a resource-limited setting where CT was unavailable.^5)^

Tension viscerothorax shares hypotension, tracheal deviation, and unilateral breath-sound loss with tension pneumothorax, creating a major diagnostic pitfall. When herniated hollow viscus rather than pleural air is the dominant space-occupying lesion, tube thoracostomy may fail to relieve mediastinal compression and may even drain enteric contents if a gastropleural communication exists. In our patient, prehospital tube thoracostomy did not improve physiology, and the combination of enteric drainage, bowel sounds in the hemithorax, and classic chest radiographic findings prompted immediate operative source control.

Imaging and diagnosis depend on the patient’s hemodynamic stability and available resources. Chest radiography can be normal or nonspecific, but intrathoracic gastric or bowel gas, loss of diaphragmatic contour, and mediastinal shift should prompt urgent consideration of diaphragmatic rupture with visceral herniation.^7)^ CT provides greater anatomic detail and may demonstrate direct or indirect signs of injury; however, even modern CT can miss isolated penetrating diaphragmatic tears.^1,2,8)^ Accordingly, diagnostic laparoscopy or thoracoscopy remains important in stable patients with persistent suspicion despite equivocal imaging. In settings where CT is unavailable, integrating mechanism, physiology, chest radiography, FAST, and the response to nasogastric decompression can justify early operative exploration when obstructive shock or peritonitis is present.^4,9)^

This case is notable for immediate multivisceral herniation after a stab wound. Penetrating injuries more often produce small defects without early herniation, leading to delayed presentation; however, larger defects may permit immediate transdiaphragmatic migration of multiple organs. Using the AAST Organ Injury Scales, the diaphragmatic laceration measuring 10 cm corresponds to grade III,^10)^ and the gastric perforation meets grade II criteria.^11)^ Standardized injury grading facilitates communication, comparison, and audit, and the revised Organ Injury Scales underscores the importance of correlating operative and imaging findings in trauma assessment.^12,13)^

The precise mechanism of the gastric perforation warrants caution. The large through-and-through fundal defect was compatible with primary traumatic laceration from the stab wound; however, because enteric drainage was observed from the pre-existing chest tube, an iatrogenic contribution from tube thoracostomy into a herniated stomach cannot be excluded with certainty. We have therefore interpreted the perforation cautiously and revised the title accordingly. This uncertainty is clinically important because blind chest tube insertion in an unrecognized diaphragmatic herniation or gastrothorax may injure the stomach and worsen pleural contamination.

The prolonged timeline before definitive operative source control (approximately 12h from injury) and the large burden of pleural enteric contamination (2.5L) likely increased the risk of pleural sepsis, gastropleural fistula, empyema, and respiratory morbidity. These features strengthen the practical message of the case: early suspicion and rapid source control are critical, particularly in resource-limited settings in which advanced imaging is not available and where inappropriate pleural drainage may exacerbate contamination.

Management of acute TDI centers on the reduction of herniated contents, repair of associated visceral injuries, nonabsorbable closure of the diaphragm, and thoracic drainage.^1–3)^ Concurrent intrathoracic gastric perforation is uncommon but carries a major septic risk; therefore, prompt lavage, drainage, and definitive source control are essential.^14)^ Most penetrating gastric perforations can be treated with primary repair when the tissue is viable, and recent series have shown this to be effective.^15)^ Strengths of this report include the clear physiologic sequence, prompt recognition using bedside examination plus CXR/FAST, and early nasogastric decompression. Limitations include the absence of CT imaging, lack of a post-NGT CXR, limited documentation from the referring center, and inability to determine with certainty whether the gastric perforation was purely traumatic or partly iatrogenic. Nonetheless, the favorable outcome in this resource-limited setting reinforces that classical clinical signs, plain radiography, early NGT decompression, and timely surgery can be lifesaving.

## CONCLUSIONS

Tension viscerothorax should be suspected after penetrating thoracoabdominal injury when obstructive shock persists despite tube thoracostomy, particularly when bowel sounds or enteric drainage are present. In the absence of CT, careful clinical assessment supported by chest radiography, FAST, and early nasogastric decompression can expedite definitive surgery and improve outcomes.

### Patient perspective

Following the injury, I experienced severe respiratory distress. The recovery after my abdominal surgery was faster than I anticipated, and I was grateful to begin eating and walking within just a few days.

## DECLARATIONS

### Availability of data and materials

The data that support the findings of this study are available from the corresponding author upon reasonable request.
